# Invasive *Pasteurellosis* of the Central Nervous System - How much can we see on a CT?

**DOI:** 10.1590/0037-8682-0592-2019

**Published:** 2020-04-22

**Authors:** Ana Carolina Freitas Ferreira, Vanessa Barcelos

**Affiliations:** 1 Serviço de Medicina Interna, Hospital do Divino Espírito Santo de Ponta Delgada, São Miguel, Açores, Portugal.

**FIGURE 1:**
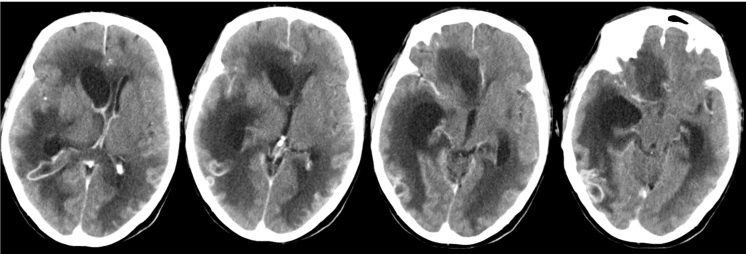
Contrast-enhanced CT scan: Intracerebral lesions involving the deep callus and white matter, especially on the right side. There is a diffuse sulcal leptomeningeal enhancement and on the ventricular contour and multiple lesions with temporo-occipital nodular enhancement. These aspects reflect the presence of brain abscesses and meningitis aggravated with ventriculitis and hydrocephalus.

A 59-year-old asthmatic man was admitted to our Emergency Department after a 48-hour history of headache, vomiting, and acute altered mental status. At admission, he had a Glasgow Coma Scale score of 6, fever, and generalized spasticity. Daily contact with dogs was reported. Laboratory test results were notable for high levels of C-reactive protein and procalcitonin. A cranial computed tomography (CT) scan revealed right occipital vasogenic edema. A lumbar puncture was performed, which showed purulent cerebrospinal fluid (CSF) with polymorphonuclear pleocytosis (more than 1000 cells/µL), glycopenia and increased protein levels. A diagnosis of bacterial meningitis was assumed, and he was transferred to the intensive care unit under sedation; invasive mechanical ventilation; and empirical therapy with ceftriaxone, ampicillin, and vancomycin. *Pasteurella multocida* was isolated from the patient’s blood, CSF, and tracheobronchial aspirate. Despite optimal antibiotherapy, his clinical condition worsened. A cranial CT-scan showed multiple abscesses, signs of ventriculitis, and cerebral infarction (Figure 1). The patient died 30 days after hospital admission.

This case describes an unusual form of *Pasteurella multocida* infection, an organism belonging to the normal oropharyngeal flora of domestic or wild animals^1^ ^-^ ^2^. Soft tissue infections after a bite or scratch are the most common forms of this condition. However, when other systems are affected, the result can be severe disease, such as meningitis or bacteremia^3^. Early identification of this etiology through careful review of the patient’s clinical history is crucial for the establishment of recommended antibiotic therapy.
